# Stem Cells behind the Barrier

**DOI:** 10.3390/ijms140713670

**Published:** 2013-06-28

**Authors:** Michael Cangkrama, Stephen B. Ting, Charbel Darido

**Affiliations:** 1 Epidermal Development Laboratory, Department of Medicine, Central Clinical School, Alfred Hospital and Monash University, Prahran VIC 3004, Australia; E-Mail: mcan6@student.monash.edu; 2 Stem Cell Research Group, Australian Centre for Blood Diseases, Central Clinical School, Alfred Hospital and Monash University, Prahran VIC 3004, Australia; E-Mail: stephen.ting@monash.edu

**Keywords:** stem cell, epidermis, differentiation, proliferation, barrier, cancer, asymmetric cell division, epigenetic, microRNA, transcription factor

## Abstract

Epidermal stem cells sustain the adult skin for a lifetime through self-renewal and the production of committed progenitors. These stem cells generate progeny that will undergo terminal differentiation leading to the development of a protective epidermal barrier. Whereas the molecular mechanisms that govern epidermal barrier repair and renewal have been extensively studied, pathways controlling stem cell differentiation remain poorly understood. Asymmetric cell divisions, small non-coding RNAs (microRNAs), chromatin remodeling complexes, and multiple differentiation factors tightly control the balance of stem and progenitor cell proliferation and differentiation, and disruption of this balance leads to skin diseases. In this review, we summarize and discuss current advances in our understanding of the mechanisms regulating epidermal stem and progenitor cell differentiation, and explore new relationships for maintenance of skin barrier function.

## 1. Introduction

The mammalian skin comprises the epidermis, a stratified squamous epithelium, and an underlying matrix-rich connective tissue, the dermis. The epidermis is composed of pilosebaceous units consisting of hair follicle (HF), sebaceous glands and the surrounding interfollicular epidermis (IFE). During embryonic development, the epidermis originates from a single layer of ectodermal cells that will undergo a stratification process allowing the formation of the different layers of differentiated cells required to fulfill its barrier function in adulthood. In addition to the epidermis’ role to combat harmful environmental toxins, it also protects the body against water loss [1]. To maintain the skin integrity and homeostasis, epidermal repair and renewal are essential. These processes are achieved by epidermal stem and progenitors cells, which are instructed by multiple mechanisms to execute programs of terminal differentiation. The epidermis has two sources of stem and progenitor cells: the IFE, which ensures tissue renewal in the absence of injury, and the HF bulge where multipotent stem cells are activated at the start of a new hair cycle and upon wounding to provide cells for HF and epidermal regeneration and repair.

Epidermal stem cells at the basal layer strongly adhere to their underlying basement membrane and ensure the lifelong maintenance of the epidermis by their inexhaustible capacity to self-renew and to differentiate into all lineages of skin epidermis [2]. These stem cells divide asymmetrically relative to the basement membrane to maintain one daughter cell with stem cell properties and the other daughter cell committed to a progenitor differentiation fate [3]. The slow-cycling stem cells support clonal units of the more rapidly cycling committed progenitors cells, which share a similar pattern of asymmetric self-renewal in which the balance between proliferation and differentiation is achieved through stochastic fate choice [4]. In their normal environment, progenitors maintain and expand the cellular pool through a combination of asymmetric cell division, symmetrical self-renewal and symmetrical differentiation divisions [5]. The proliferating progenitors, which are often referred to as transient amplifying (TA) cells, will withdraw from the cell cycle to migrate and differentiate into the distinctive suprabasal spinous, granular and stratum corneum layers of the epidermis [6]. At the outermost stratum corneum layer, the terminally differentiated cells are sealed together to form a waterproof barrier and are eventually shed from the skin surface. These cells mark the boundary between the inner, metabolically active strata and the outer, inert layer consisting of dead cells with degenerate nuclei and absent intracellular organelles. The mechanisms controlling epidermal stem cell self-renewal and differentiation leading to skin barrier maintenance are still poorly understood. Insights into these mechanisms will eventually address clinical scenarios where restoration of the skin barrier is required, and new therapeutic options for uncontrolled epidermal cell proliferation, *i.e.*, skin cancers.

Functional and lineage tracing assays were the founders for the identification and isolation of stem cells. The slow cycling property of epidermal stem cells, which conserves their proliferative potential and minimizes DNA replication-related errors, allow their labeling by continuous administration of nucleotide analogs such as [^3^H] thymidine or bromodeoxyuridine (BrdU) for a prolonged period. The rapidly cycling TA cells dilute the label, however the stem cells that divide less frequently during the chase period retain the label and hence, are functionally referred to as label-retaining cells (LRC). The detection of interfollicular LRC confirmed the existence of a discrete population of quiescent IFE stem cells [7]. To genetically track epidermal progenitor cells in adult mice, a transgenic mouse model expressing histone 2B-green fluorescent protein (H2B-GFP) under the control of an inducible tetracycline-regulated enhancer element has been used to mark a sample of cells and their progeny. During pulse chase experiments, most epidermal cells diluted out the H2B-GFP label during division whereas the majority of H2B-GFP LRC showed expression of the stem cell marker, CD34 [8]. Stem cells in the HF bulge have been purified based on the expression of several surface markers including Lgr5 [9], Keratin (K)-15 [10], CD34 and α6-(CD49f) integrin, and with purification by fluorescence-activated cell sorting (FACS) of K14-GFP expressing cells [11,12]. For human IFE stem cells, a range of markers has been described, including high surface expression of β1-integrins [13], α6-integrin, Delta1 and low expression of CD71 [14], desmoglein 3 [15], and the EGF receptor 1 [16]. Also, the melanoma-associated chondroitin sulfate proteoglycan (MCSP) has been defined as a reliable stem cell marker that is undetectable in other basal cells of the human epidermis [17]. Although each of these different procedures purifies slightly different cell populations, their data are in good agreement, and gradients of marker expression are consistent with gradients of “stemness”, as opposed to the existence of discrete stem and TA cell populations [18]. These findings shape the framework enabling researchers to interpret results from interdisciplinary approaches to learn more about epidermal stem cells.

## 2. Stem Cell Differentiation and Skin Homeostasis

Several factors regulate stem cell differentiation in the IFE, which allow the generation of epidermal progenitors. Progeny that become committed to differentiation go through several rapid divisions, then stop dividing and move out toward the surface of the skin during terminal differentiation. This coordinated program of cell differentiation is initiated in the basal layer, where the destination of stem and TA cells is decided. In response to external stimuli, spatial and temporal activation of epidermal stem cells regulate the rate of basal cell proliferation as a prelude to terminal differentiation. Within this context, some fundamental questions in epidermal homeostasis remain—What factors determine whether the stem cell proliferates or stays quiescent? What decides whether a progenitor cell differentiates or expands? Which pathways are followed through to terminal differentiation? And what are the consequences of disrupting these pathways?

Quiescent adult stem cells reside in specialized niches where they become activated to proliferate and differentiate during tissue homeostasis. During normal epidermal homeostasis, each stem cell population feeds a restricted number of differentiated lineages. However, in response to injury or genetic manipulation the different pools of stem cells demonstrate multi-lineage differentiation ability [19]. Genetic lineage-tracing experiments in adult mice have shown that bulge cells repair the wound only transiently; raising the possibility that long-term stem cells involved in tissue repair may be present in the epidermis [20]. In pathological conditions such as skin injury and barrier loss, quiescent stem cells near the affected wound would be expected to replace activated stem cells lost to the reparative proliferation-differentiation process. This would prevent the active stem cell pool from becoming exhausted and protect against accumulating potentially tumorigenic mutations during DNA replication. It was shown that proliferative stimuli induce exiting HF stem cells from their quiescent state, which might contribute to the initial proliferative burst of epithelial cells in the HF, followed by the exhaustion of the HF stem cell population [21]. This highlights the importance of an appropriate balance between the proliferative and quiescent states, which is critical to maintaining a functional stem cell compartment. It is therefore tempting to speculate that in some circumstances the stem cell pool becomes exhausted and this would result in a barrier defect. The combination of these reciprocal backup systems would provide a robust mechanism to ensure a high rate of physiological self-renewal as well as flexible damage repair, after which the original hierarchy could be reestablished [22]. Loss of function studies attributed regulatory mechanisms of stem cell quiescence to several factors such as NFATc1 in HF [23] and Lrig1 in IFE [24]. However, the implications of these factors in barrier maintenance and the molecular mechanisms that sense pathological conditions to activate the quiescent stem cells remain to be elucidated.

## 3. Asymmetric Cell Division and Epidermal Barrier Function

Polarized intra-cellular protein segregation and protein complexes regulating mitotic spindle positioning govern asymmetric cell division and self-renewal in stem cells [3,25]. The planar cell polarity pathway has also been shown to regulate asymmetric stem cell division, morphogenesis and differentiation in the skin [25–27]. In the context of epidermal stem and progenitor cells, division orientations are defined by the long axis of the mitotic spindle in reference to the basal layer, where perpendicular and parallel spindle axes respectively, define an asymmetric and symmetric cell division. Although these two modes of stem cell division control the balance between proliferation and differentiation, terminal epidermal differentiation and stratification is dependent on asymmetric epidermal stem cell divisions. This is exemplified during development at embryonic (E) day 14 where perpendicular stem cell divisions lead to stratification and terminal differentiation [25,28]. One gene governing epidermal asymmetric cell division and stratification is p63, as cell divisions in the p63-null epidermis of E18.5 embryos were symmetric, *i.e.*, parallel to the basal layer and exhibited defective epidermal stratification [29,30]. In adult mice, homeostasis of stratification also seems to be dependent on perpendicular divisions because most divisions in both adult epidermis (about 85%) and tongue (about 65%) still occurred in this fashion, even though overall mitoses waned considerably once animals reached full size [3]. Mechanistically, and compared to control littermates, apical accumulation of LGN, an important orchestrator of spindle orientation and planar cell division, was not detected in the p63-null epidermal keratinocytes. Furthermore, studies with *in vivo* LGN knockdown using epidermal-specific lentiviral RNA interference showed impaired asymmetric cell divisions, stratification and barrier function with resultant newborn LGN-deficient pups dying several hours after birth due to severe dehydration [31]. Transgenic mice of TAp63α isoform showed that p63 is required for the commitment to stratification in part by induction of the AP-2 transcription factor family [32]. AP-2alpha knockout mice did not present with barrier defects, however AP-2alpha and gamma double knockout mice showed a block in terminal differentiation and barrier impairment. At the molecular level, AP-2 factors were shown to co-operate with Notch signaling to orchestrate terminal differentiation in skin epidermis [33], thereby linking the p63 pathway to Notch signaling in epidermal homeostasis. Moreover, asymmetric cell division was shown to promote Notch signaling that further stimulated suprabasal terminal differentiation [31] and importantly, loss of Notch in the skin disrupts the epidermal barrier and leads to increased proliferation and spontaneous tumor development [34,35].

## 4. Terminal Differentiating Factors and Stem Cell Differentiation

**Figure 1:**
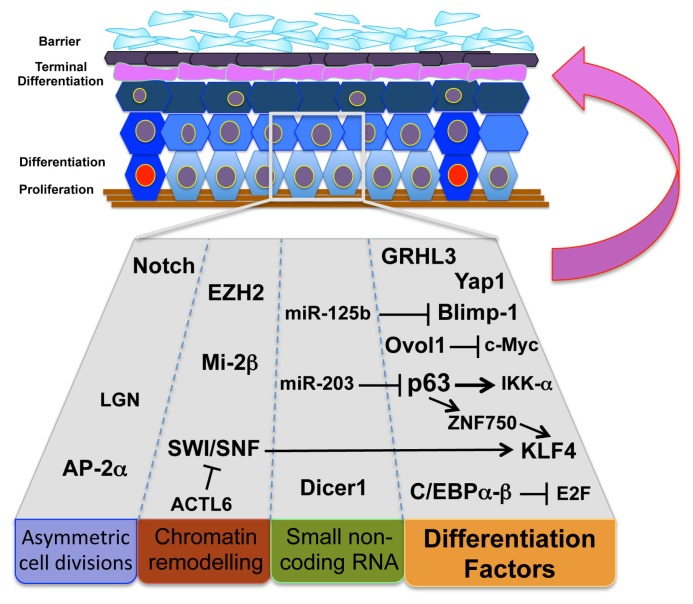
Processes of stem and progenitor cell differentiation involved in skin barrier function. Components regulating asymmetric cell divisions, chromatin remodeling, small non-coding RNA and differentiation factors initiate differentiation programs at the stem cell niche that will progress to terminal differentiation leading to epidermal barrier establishment and maintenance.

Gene deletion studies with an impaired skin barrier phenotype have shown numerous mechanisms contribute towards normal epidermal differentiation (Figure 1). Several transcription factors, including p63 [36], Klf4 [37,38], Ovol-1 [39], C/EBP-α/β [40], Blimp-1 [41], Yap1/TEAD [42], and Grhl3 [43] have been implicated in the regulation of terminal differentiation and barrier formation, and recent findings suggest these factors function at the level of the epidermal stem cell.

p63, which regulates epidermal development and stem cell self-renewal as discussed above, is required for the initiation of the stratification program via asymmetric cell division and epidermal differentiation [29,30,44]. p63 is expressed in all the metabolically active layers of human epidermal tissue, where it is required for induction of both early and late epidermal differentiation genes [30]. The defective single layer of epithelial cells covering p63-deficient mice fails to provide barrier function at birth, resulting in early postnatal lethality due to severe dehydration [36]. Koster *et al*. propose a role for p63 in triggering basal cells to switch from proliferation to terminal differentiation [29] through the induction of IκB kinase-α (IKK-α), a putative regulator of terminal differentiation. Interestingly, IKK-α deficient mice also manifest epidermal barrier defects where neonates die early at birth due to severe dehydration [45]. In a separate study, p63 was also shown to induce a later epidermal differentiation gene program via the nuclear factor, ZNF750, which regulates the differentiation specific transcription factor, KLF4 [46]. Indeed, mice deficient for Klf4 die shortly after birth due to loss of terminal differentiation, skin barrier defects and severe dehydration [38]. These mice show increased cell proliferation and enhanced skin tumorigenesis, highlighting the importance of Klf4 in maintaining the epidermal proliferation-differentiation balance [47]. Supporting evidence that p63-induced epidermal differentiation is initiated at the stem cell comes from studies involving the predominant p63 isoform, ΔNp63α where gain of ΔNp63α function results in skin cancer. Mechanistically, ΔNp63α targets a SNF2 chromatin remodeling protein, LSH (lymphoid-specific helicase) with resultant bypass of oncogene-induced senescence and subsequent survival and proliferation of Keratin15-positive epidermal stem cells [48].

The transcription factor Ovol-1 is expressed in embryonic epidermal progenitor cells that are transiting from proliferation to terminal differentiation. Ovol-1 mutant mice are born with a defective epidermal barrier and die perinatally within the first 2 weeks [39]. Ovol-1 deficient epidermis fails to restrict the proliferative potential of progenitor cells, and cultured keratinocytes fail to efficiently undergo growth arrest in response to extrinsic growth-inhibitory signals. The mechanism by which Ovol-1 regulates proliferation exit of committed epidermal progenitors is through repressive binding of the promoter of the proto-oncogene, c-Myc [49]. In contrast, c-Myc balances the processes of stem cell self-renewal, proliferation and differentiation in adult skin by binding to transcriptional regulators that induce epidermal differentiation complex (EDC) genes. Another method by which both Klf4 and Ovol-1 induce terminal differentiation is via their respective recruitment to the EDC promoter regions to form negative regulatory feedback loops to repress c-Myc [50].

The transcription factors C/EBP-α and -β are co-expressed in basal keratinocytes, and are coordinately increased as keratinocytes exit the basal layer and undergo terminal differentiation. Mice lacking both C/EBP-α and -β in the epidermis showed increased proliferation of basal cells and impaired commitment to differentiation, which manifest as a defective epidermal barrier function and perinatal death within eight hours of birth. Mechanistically, C/EBP interaction and repression of the transcription factor E2F was required for restricting the epidermal stem cell compartment and inhibiting IFE proliferation. When C/EBP-α and -β were lost, the stem cell-specific gene expression signatures were upregulated in the epidermis together with a significant decrease in epidermal stem cell quiescence [51]. A role for C/EBP factors in suppressing stem cell gene expression is crucial in the coupling between cell cycle exit and the commitment of epidermal differentiation that could also provide a basis for their ability to modulate epidermal cancer susceptibility [51].

In mice, the transcriptional repressor Blimp-1 has been proposed to regulate sebaceous gland homeostasis via unipotent progenitor cells within the sebaceous glands of the skin. Loss of Blimp-1 leads to enhanced stem cell activity from the hair follicle bulge, suggesting that when normal sebaceous gland homeostasis is perturbed, multipotent stem cells in the bulge can be mobilized to correct this imbalance [52]. However, these data are not in agreement with Blimp-1 expression in humans where it is a marker of terminal differentiation throughout the skin and its appendages, but not a progenitor marker for sebocytic cells [53]. More specifically, within the IFE, the exclusive localization of Blimp-1 expression to the granular layer of both developing and adult skin suggests a central function in skin barrier homeostasis [53]. In keeping with these observations, conditional K14-Blimp-1 deletion disrupts the maturation of granular cell to corneocytes and delayed epidermal barrier formation [41]. Further investigations will be necessary to uncover the association between Blimp-1 and epidermal barrier function from a skin stem cell perspective.

The transcriptional effector of the Hippo signaling pathway Yes-associated protein (YAP1) is highly expressed in epidermal basal progenitors where it is required to balance stem cell self-renewal, proliferation and differentiation [42,54,55]. Yap1-deficient mice express an impaired epidermal barrier dying within hours after birth. Examination of these embryos revealed thinner, fragile skin, absence of epidermal tissue covering the distal part of the limbs and inhibited proliferative potential as shown by a reduction in the number of colony-forming progenitors. Elevated nuclear YAP1 functions as a transcriptional coactivator through interaction with TEA domain (TEAD) transcription factors to expand the epidermal stem cell compartment, whereas during epidermal differentiation YAP is localized to the cytoplasm suggesting that regulation of YAP1 subcellular localization might be important for the switch between proliferative and terminally differentiating compartments [42]. Gain-of-function experiments have also shown that activation of Yap1 induces expansion of an undifferentiated stem/progenitor cell population in the IFE and leads to tumor formation [54]. An intriguing parallel is the resemblance between the hair follicle evagination phenotype seen in both the Yap1-transgenic and the Dicer epidermal-conditionally targeted mice where Dicer encodes a core component of the microRNA (miRNA) processing machinery [56]. Whether YAP1 blocks miRNA expression or conversely, epidermal Dicer deletion leads to increase YAP1 levels remains to be determined.

The RNA exosome participates in the degradation and processing of a wide range of RNA molecules. The EXOSC9 subunit of the exosome has been recently shown to maintain the self-renewal of the stem cell pool in the skin. Epidermal EXOSC9 maintains the progenitor state by directly binding to and degrading mRNAs that encode for the GRHL3 transcription factor, a major inducer of epidermal differentiation [57]. The authors suggest that during differentiation, several subunits of the exosome are downregulated, which results in the stabilization of Grhl3 transcripts, leading to higher levels of GRHL3 protein and allowing for transcription of differentiation genes. Loss of the exosome promotes the differentiation of stem cells into progenitors resulting in loss of epidermal tissue over time. Furthermore, preventing the increase in Grhl3 levels in EXOSC9 knockdown cells can rescue EXOSC9 regulated genes and tissue phenotype suggesting that maintaining GRHL3 at low levels is critical for preventing premature differentiation [57]. However, this finding is striking, considering that factors involved in recruiting the exosome to Grhl3 transcripts to promote self-renewal in progenitor cells remain unknown. Grhl3 expression is slightly detectable in basal cells and is significantly increased in the differentiated suprabasal layers [58,59]. The GRHL3 factor maintains epidermal terminal differentiation and Grhl3-deficient mice die at birth due to failure of skin barrier formation and severe dehydration [60–62]. Conditional ablation of Grhl3 in adult mice results in increased proliferation rates and the development of spontaneous tumors [63]. In addition, targeting of Grhl3 by a microRNA-21-proto-oncogenic network in human keratinotyes leads to the development of skin and head & neck cancers [63]. Thus, the function of miRNA-21 in different epidermal compartments and its regulation of differentiation factors during epidermal proliferation merits ongoing investigations as to whether Grhl3 and miRNA-21 are active in epidermal stem cells.

## 5. MicroRNA Regulation of Epidermal Homeostasis

Increasing evidence suggests that small non-coding, micro RNAs (miRNAs) are important players in epidermal stem cell self-renewal and epidermal morphogenesis and differentiation. The functional importance of these small RNAs in skin development is underscored by the phenotype of conditional deletion of the gene that encodes the miRNA-processing enzyme, Dicer1 in skin epithelium, where epidermal morphology is disturbed. The skin barrier in Dicer1 conditional null mice is severely compromised and the neonates died early at birth from dehydration as determined by significant weight loss [56]. An extension of this work is the identification of specific miRNAs shown to have a functional role in epidermal homeostasis.

One of these miRNAs is miR-125b, which is preferentially expressed in epidermal stem cells to balance self-renewal and early lineage commitment. Sustained epidermal expression of miR-125b via the K14-promoter showed that transition from “stemness” to committed progenitor is markedly retarded in both an inducible *in vivo* system and *in vitro* gain- and loss-of-function assays. The effect of miRNA-125b to enhance epidermal stemness at the expense of differentiation was partially attributed to targeting of the epidermal terminal differentiation factor, Blimp-1 [64].

The miR-203 was shown to target and negatively regulate suprabasal expression of basal genes, thereby acting as a switch between proliferation and differentiation [65]. miRNA-203 restricts the proliferative potential of epidermal stem cells, as shown by comparison of the clonogenic capacity of primary mouse keratinocytes. Wild-type keratinocytes formed typical holoclones composed of small, undifferentiated cells capable of long-term passage. By contrast, keratinocytes from transgenic mice overexpressing miRNA-203 under the control of K14-promoter produced mostly paraclones, composed of large, flattened cells. The authors identified that the restrictive effect of miR-203 on epidermal stem cells functioned through targeting the p63 3′UTR, which defined a molecular boundary between proliferative basal progenitors and terminally differentiating suprabasal cells. Furthermore, the depletion of basal stem cells in conditional K14-miR-203 overexpressing keratinocytes bore a resemblance to the p63 null epidermis [65]. Future identification of other miRNAs and their respective target genes involved in epidermal stem and progenitor cell differentiation would potentially identify new and novel pathways important for skin barrier function.

## 6. Chromatin Remodeling Complexes and Differentiation Programs

The functional effects of epigenetic regulation in stem cells could be summarized as limiting or enhancing accessibility of gene regulatory regions with resultant expression of stem or differentiation programs. Molecularly, the epigenetic machinery can function at the level of DNA-protein interactions, covalent DNA and histone modifications, ATP-dependent chromatin remodeling, and nuclear sub-compartmentalization of the transcription machinery [66]. Many studies have now demonstrated the crucial role of epigenetic and histone modifications in the regulation of stem cell proliferation and differentiation [67–72]. Not surprisingly, epigenetic regulation of stem progenitor cells requires co-operative maintenance of the progenitor state with tight suppression of differentiation genes, as premature expression of the latter can abolish proliferative capacity and trigger cell death.

The SWI/SNF complex is a chromatin remodeling complex that functions to destabilize histone-DNA interactions, resulting in an open chromatin state and leading to transcriptional activation. The SWI/SNF complex consists of the catalytic ATPase subunits Brg1 and Brm as well as 11 regulatory subunits. Conditional deletion of Brg1 in the skin shows severe defects of epidermal barrier function with alterations in the structure of the cornified layer, but normal basal, spinous, and granular layers. While Brm-deficient mice develop normally and do not have any skin defects, the epidermis of double Brg1/Brm-deficient mice in comparison to Brg1-deleted skin, demonstrated increased morphological abnormality of suprabasal layers and subsequently, a more severe skin barrier defect suggesting a functional redundancy for Brm in the epidermis [71].

ACTL6a (actin-like 6a), an epigenetic repressor of differentiation, was recently shown to be significantly downregulated during epidermal differentiation [70]. The authors suggest that ACTL6a maintains the undifferentiated progenitor state by opposing the SWI/SNF chromatin-remodeling complex, which enabled activation of Klf4 and other epidermal differentiation genes. Furthermore, loss of the terminal differentiation factor KLF4 significantly compensated for the defects caused by ACTL6a depletion in progenitors. Conditional deletion of ACTL6a in mouse epidermis abolished epidermal progenitor function, leading to cell-cycle exit, terminal differentiation, and ultimately hypoplasia, followed by epidermal tissue loss [70]. Newborn ACTL6a-null animals die within only a few hours after birth and are characterized by thin-appearing skin with epidermal erosions and the induction of differentiation proteins in the basal layer, without a significant increase in apoptosis.

The chromatin remodeler Mi-2β is essential for epidermal stem cell function during early embryogenesis. Mi-2β and its associates have a crucial role in restructuring a chromatin environment permissive for the gene expression profile that drives epidermal progenitor differentiation. Using a Mi-2β LacZ-reporter mouse, the Mi-2β expression was shown to initiate at E10.5 in the ventral skin and from E14 onwards in the dorsal skin. Assessment of Mi-2β knockout mice showed defects in terminal differentiation and impairment of barrier function initially on the ventral side of animals [72]. The impact of Mi-2β depletion during early skin development appears to alter the properties of the emerging epidermal precursors by a reduction in both self-renewal capacity and maintenance of cell differentiation. This model suggests that extended self-renewal capacity is actively conferred on a progenitor with more limited proliferative capacity, and that abnormal differentiation of the skin can be attributed to a lack of plasticity in the basal epidermal progenitor [72].

Ezh2, a member of the polycomb repressor complex (PRC2), is important in mediating the recruitment of PRC1 to repress gene expression either by chromatin compaction or by interfering with the transcription machinery. Ezh2 is expressed in epidermal progenitors and controls proliferative potential of basal progenitors by repressing the Ink4A-Ink4B locus. This function diminishes concomitant with differentiation, and progressive Ezh2 loss leads to recruitment of AP1 transcriptional activator to the structural genes that are required for late-stage epidermal differentiation and barrier acquisition during skin development [68]. Conditional loss of Ezh2 in the skin impaired proliferation and induced the premature differentiation of the epidermis during embryonic development. The barrier acquisition was accelerated in the mutant embryos as manifested functionally in the dye exclusion assay. This phenotype disappeared post-natal and did not impair subsequent development: a possible explanation is that Ezh2 expression wanes after birth and that either the Ezh2 paralog, Ezh1 or alternative mechanisms are activated to control homeostasis in the adult epidermis [68].

## 7. Differentiation Defects and Epidermal Cancers

Defective regulation of epidermal differentiation pathway components is associated with cancer initiation and progression [63,73,74]. Reprogramming and cell fate changes lead to common and rare human skin disorders, including psoriasis, atopic dermatitis, ichthyosis vulgaris, and epidermal cancers [75–79]. Although still debated, one thought is that a cancer stem or initiating cell is the cell of origin of both basal cell carcinomas (BCC) and squamous cell carcinomas (SCC). In this so-called cancer stem cell hypothesis, a rarely dividing epidermal stem cell with its long-lived nature accumulates multiple genetic hits before finally overcoming cell cycle control with resultant neoplastic growth. Alternatively, non-stem progenitor cells may exhibit genetic alterations that interfere with differentiation whilst acquiring self-renewal characteristics that combine to bestow cancer-initiating properties. It is crucial to reinforce that factors such as Klf4, Grhl3, Notch1, p63 and others, are defined tumor suppressors, which supports the hypothesis that stem cell differentiation factors are critical to maintain epidermal homeostasis.

Although epidermal LRCs are capable of expanding during skin tumor promotion [80], a stem cell gene expression profile of human SCC did not reflect a simple expansion of the normal stem cell compartment. Using a panel of stem cell markers and different heterogeneous SCC lines tumor development was dependent on pathways that control epithelial homeostasis and stem cell quiescence, particularly in the basal cell layer, where a positive effect on proliferation and inhibition of differentiation was noted [81]. [^3^H] Thymidine-labeling has shown that epidermal stem cells scarcely entered mitosis and remained in the basal layer upon 12-O-tetradecanoylphorbol 13-acetate (TPA) treatment, whereas the proliferating cells dislocated rapidly from the basal layer during terminal differentiation [82]. Furthermore, LRCs of both HF and IFE retained carcinogen-DNA adducts, and even after ablation of cycling cells in the epidermis with a chemotherapeutic drug prior to 7,12-dimethylbenz[a]anthracene (DMBA) treatment, the rate of carcinoma formation was unchanged, indicating that tumor initiation occurred in quiescent stem cells rather than rapidly proliferating TA cells [83].

Interestingly, genetic mouse models have suggested that BCCs may not derive from HFs but may originate from the IFE [84]. Conversely, studies of SCCs, long believed to have originated only from the IFE, are providing more evidence that these skin cancers may also be attributed to HF bulge stem cells [85]. Whether this spectrum directly translates to equivalent human cancers remains to be seen. As identification of the cancer-initiating cell in human skin carcinomas continues to be investigated, it is anticipated that the origin of both BCCs and SCCs will involve a stem cell or progenitor cell with acquired stem cell-like properties.

## 8. Conclusions

Recent evidence is emerging to highlight the importance of the stem-progenitor proliferation-differentiation balance in continuously renewing systems. A single population of dividing cells maintains the epidermal homeostasis in normal conditions [86]. In pathological circumstances such as barrier defects, the proliferation rate should increase to correct the damage. Mechanistically, and not mutually exclusive, this may occur through activation of quiescent epidermal stem cells; or cells that are committed to terminal differentiation functionally reverting to stem cells [87]; or increasing the proportion of symmetrical cell divisions resulting in two proliferating daughter cells. All of these potential mechanisms would probably co-involve repression of differentiation factors.

Initiation of epidermal differentiation requires the establishment of asymmetric cell divisions and terminal epidermal differentiation results in functional skin barrier formation. A unifying hypothesis is that maintenance of asymmetric cell divisions and subsequent programs of differentiation are crucial for terminal differentiation and epidermal barrier function. Epidermal barrier defects could therefore be considered phenotypic of defective epidermal stem cell proliferation-differentiation homeostasis.

Loss of the asymmetric cell divisions leads to two identical daughter cells with either stem cell properties or committed progenitors properties. Both situations are expected to cause epidermal barrier defects. In the case of epidermal stem cell deficiency, progenitors lead to terminal differentiation to renew the barrier, which will eventually be lost when the proliferating progeny is exhausted. This hypothesis is supported by the function of p63 as a lineage-specific determinant of the proliferative capacity in stem cells, where p63-deficient stem cells undergo a premature proliferative rundown [44]. On the other hand, in the case of committed progenitor deficiency, epidermal stem cells increase in number with correlative increased proliferation but incomplete differentiation resulting in a defective epidermal barrier. In certain conditions, aberration of asymmetric cell divisions and subsequent stem cell depletion or expansion can also lead to various epidermal anomalies including cancers [88].

Future studies aim to decipher how perturbation of terminal differentiation factors may mediate regulation of epidermal stem cells. These insights are expected to provide novel treatment strategies for skin disorders and to translate into the medical era of tissue regeneration and repair.
